# Dosimetric impact of different bladder and rectum filling during prostate cancer radiotherapy

**DOI:** 10.1186/s13014-016-0681-z

**Published:** 2016-08-02

**Authors:** Zhi Chen, Zhaozhi Yang, Jiazhou Wang, Weigang Hu

**Affiliations:** Department of Radiation Oncology, Fudan University Shanghai Cancer Center, 270 DongAn Road, Shanghai, 200032 China

**Keywords:** Cone-beam CT, Dose tracking, Prostate cancer

## Abstract

**Background:**

The aim of this study was to analyze the influence of volumetric changes of bladder and rectum filling on the 3D dose distribution in prostate cancer radiotherapy.

**Methods:**

A total of 314 cone-beam CT (CBCT) image data sets from 19 patients were enrolled in this study. For each CBCT, the bladder and rectum were contoured and volume sizes were normalized to those on their original CT. The daily delivered dose was recalculated on the CBCT images and the doses to bladder and rectum were investigated. Linear regression analysis was performed to identify the mean dose change of the volume change using SPSS 19.

**Results:**

The data show that the variances of the normalized volume of the bladder and the rectum are 0.13–0.58 and 0.12–0.50 respectively. The variances of V_70Gy,_ V_60Gy,_ V_50Gy,_ V_40Gy_ and V_30Gy_ of bladder are bigger than those of rectum for 17 patients. The linear regression analysis indicates a 10 % increase in bladder volume will cause a 5.6 % (±4.9 %) reduction in mean dose (*p* <0.05).

**Conclusions:**

The bladder’s volume change is more significant than that of the rectum for the prostate cancer patient. The rectum volume variations are not significant except for air bubbles, which change the shape and the position of the rectum. The bladder volume variations may cause dose changes proportionately. Monitoring the bladder’s volume before fractional treatment delivery will be crucial for accurate dose delivery.

## Background

Most radiotherapy patients with prostate cancer are treated with intensity modulated radiotherapy (IMRT). Especially volumetric modulated arc therapy (VMAT) helps to get highly conformal dose distributions for the planning target volume (PTV) while minimizing the dose given to the organs at risk and with short delivery time. However, the advantages of IMRT and VMAT are limited by daily treatment uncertainties [1–3]. Daily treatment uncertainties include daily patient setup errors and internal organ motion and deformation. Studies on prostate motion have shown that both the position and the shape of the prostate vary throughout the course of treatment [4, 5].

Currently, CBCT is a popular imaging method that provides valuable 3D information of the patient in treatment position [6–8]. In our hospital, we commissioned an Elekta Synergy VMAT accelerator with on-board kV-CBCT in 2011, which was used to improve the geometric accuracy of target localization in radiation therapy. In principle, CBCT can be used for dose calculation. Richter et al. reported small dose differences between planning CT and CBCT with 0.9 % ± 0.9 % for pelvis, 1.8 % ± 1.6 % for thorax and 1.5 % ± 2.5 % for head [9]. In our institution, Hu et al. showed the feasibility of using a region-of-interest (ROI) mapping method for accurate CBCT-based dose calculation [10].

Both rectum and bladder volumes change during the course of radiotherapy for prostate cancer. Roeske et al. observed that bladder and rectal volumes varied by ±30 % according to treatment planning computed tomography [11]. Huang et al. used CBCT to analyze the changes of the bladder and rectum during fractionated radiotherapy of the prostate. The differences in the volume and radiation dose were 44 %(±41) and 18 %(±17) for the bladder and 36 %(±29) and 22 %(±15) for the rectum [12]. During the course of image-guided radiotherapy in the post-prostatectomy, Akin et al. assessed the dose-volume parameters (V_40%_, V_50%_, V_60%_, V_65%_) for the bladder and rectum, and found the most actual doses delivered were higher than calculated in the treatment planning CT: the V_40%_, V_50%_, V_60%_ and V_65%_ of the bladder were 20 % more higher than calculated in the treatment planning CT, the V_40%_ of rectum is 8.7 % higher than the calculated in the treatment planning CT, and the V_60%_ of rectum is 59.6 % higher than calculated in the treatment planning CT, while the V_50%_ and V_60%_ of rectum did not show significant difference [13].

The purpose of this study is to track the volume and dosimetric changes in the bladder and rectum based on daily cone-beam CT for prostate radiotherapy. We hope to predict the bladder dose using the volume obtained before treatment.

## Methods

### Patient data acquisition

Nineteen prostate cancer patients were included in this study. Of these, 16 patients with stage T1-3 N0M0 had radiotherapy (RT) with total prescribed dose from 70.2 to 79.2 Gy by 1.8 Gy per fraction. Three patients with biochemical failure after prostatectomy had salvage RT with a total prescribed dose of 64.8 Gy by 1.8 Gy per fraction. For patients with radical RT, the clinical target volume (CTV) was defined as the prostate gland and seminal vesicle as visible on the CT scan. For patients with salvage RT, the CTV included the prostate and seminal vesicle bed. The planned target volume (PTV) was defined as CTV plus 5 mm in the posterior direction and 8 mm for all other directions. The rectum, bladder, femoral heads were also contoured for each patient. The patients were required to drink a glass of water about 30 mins before the acquisition of treatment planning CT under the supervision of physicians. Also, the patients were reminded to drink the same amount of water 30 mins before each fractional treatment. The VMAT plan optimization and dose calculation was carried out in the treatment planning system Pinnacle3 (Philips Pinnacle3 V8.0d, Fitchburg, WI, USA). The imaging protocol consisted of a planning CT (AcQsim CT Simulator, Philips Medical System, Cleveland, OH) and daily CBCTs (Elekta, Synergy S, XVI, Crawley, UK) acquired in treatment position. The following parameters of CT and CBCT were used for imaging: CT: 120kVp, high-resolution reconstruction (512 * 512), with a slice thickness of 5 mm; CBCT: 120kVp, FilterType ‘M’, reconstruction resolution (410 * 410), with a slice thickness of 3 mm. Each patient’s CBCT database contained 8–27 CBCTs. Most of the patient’s CBCTs were acquired once every 2 days or twice a week or twice in 3 days. One patient only had the first ten CBCTs during the treatment course. A total of 314 CBCTs are included in this study. The same physician delineated the bladders and rectums for consistency.

### Dose calculation on CBCT

To calculate the “dose of the day” and assess the dose to the bladder and rectum, the CBCTs were rigidly auto-aligned to the planning CT using RayStation treatment planning system (Version 3.0, RaySearch Laboratories AB, Stockholm, Sweden). All the original planning data including the CT data sets, RT structures, and RT plans were exported to the RayStation treatment planning system for dose recalculation. The same Synergy machine was commissioned in the RayStation and the recalculated dose distributions between the Pinnacle and RayStation were verified within 1.5 %. Due to the recalculations of the dose on CBCT were performed in the RayStation, the original dose distributions on CT were also recalculated in RayStation which used as plan dose, and comparisons were performed on the same TPS for reliability purpose. In order to use CBCT images for dose calculation, the image pixel values were converted from CBCT numbers to physical density using the ROI CT number mapping method as reported [10]. A brief description of the ROI CT number mapping method is as follows: 1) The CBCT images and the CT images of the same patient were acquired on the same day to minimize the change in patient anatomy between the two images. 2) The CT and CBCT images were registered in the RayStation TPS. 3) The ROIs were mapped from CT to the CBCT, and the mean CBCT number values of the ROIs were recorded. 4) Generate the CBCT numbers to physical density calibration curve based on the density values measured on the CT. After that, the original plan was rigidly copied to the CBCT image data set for dose recalculation.

### Dose and volume evaluation

All bladder and rectum volumes on the CBCTs were normalized to those on the original CT to track the volume changes. Additionally, the bladder and the rectum were mapped to the planning CT using MIM software version 6.1(MIM Software Inc., Cleveland, OH, USA) to visualize the deformation of the bladder and rectum. The V_70Gy_, V_60Gy_, V_50Gy_, V_40Gy_, V_30Gy_, V_20Gy_ and V_10Gy_ for the rectum, and V_70Gy_, V_60Gy_, V_50Gy_, V_40Gy_ and V_30Gy_ for the bladder were calculated respectively. The deviation of the mean dose to the bladder and rectum were also evaluated. A linear regression analysis was performed to identify the mean dose change vs. the volume change using SPSS version 19.0 (SPSS, Inc., Chicago, IL, USA). The mean doses and volumes were all normalized to their corresponding dose and volume on the original plan.

## Results

### Volume evaluation

**Fig. 1:**
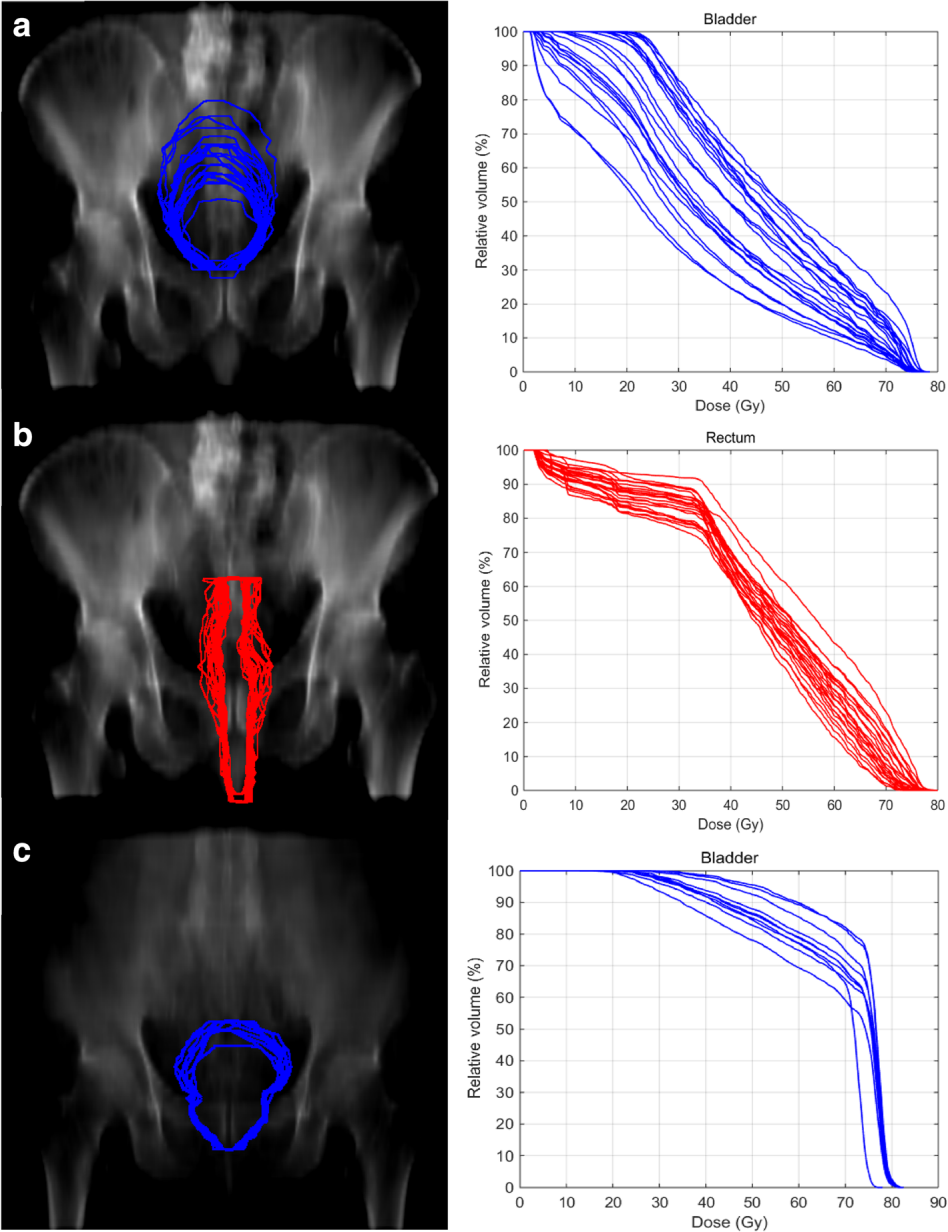
DRR of two patients. **a**–**b** show bladder and rectum contours for one patient derived from the different CBCTs projected on one DRR. The corresponded DVHs are displayed as well. **c** demonstrates different projections of the bladder contours on one DRR for a patient who had only very slight changed in his bladder filling

**Fig. 2:**
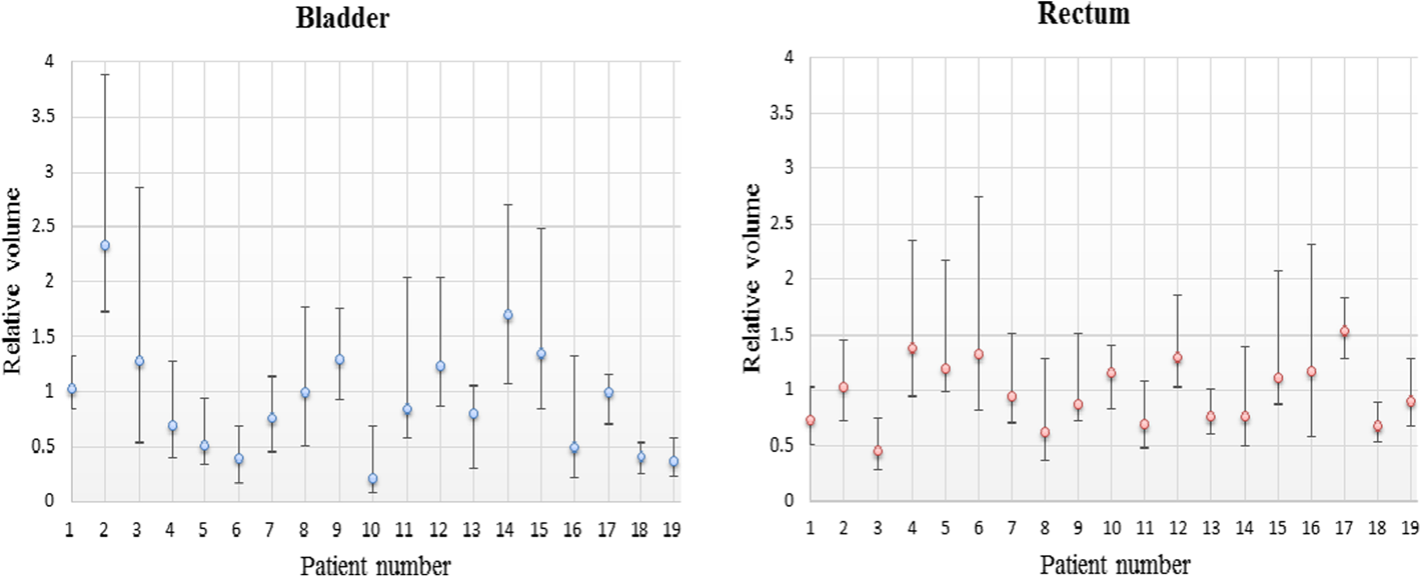
The variation of the relative volumes for the bladder and rectum

For each patient, the volumes of bladder and rectum on the CBCT were different to those on the planning CT. The deformation of the bladder and the rectum could be clearly identified. Figure 1(a)–(b) show bladder and rectum contours for one patient derived from the different CBCTs projected on one DRR. The corresponding DVHs are displayed as well. Figure 1(c) demonstrates different projections of the bladder contours on one DRR for a patient who had only very slight changed in his bladder filling. The result from the nineteen patients show that the variance of the normalized volume (normalized to corresponding OARs in planning CT) of the bladder is 0.13–0.58 and the rectum is 0.12–0.50. Figure 2 shows the variance of the bladder and the rectum for all the patients. Among the 19 patients, 13 patients’ variances of volumes for the rectum are smaller than those of the bladder. But in terms of absolute volume, the variances of the rectum are all smaller than the bladder.

### Dose evaluation

**Fig. 3:**
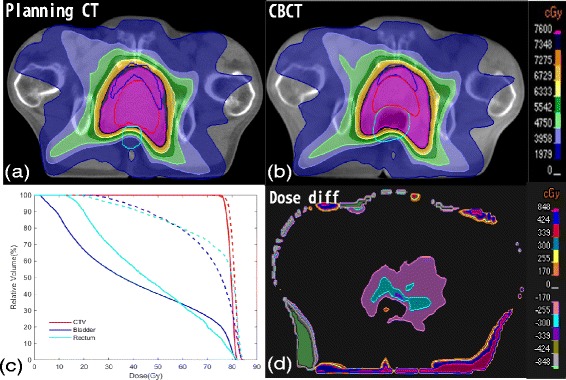
**a**–**b** represent dose distribution of the same transversal plane of the same patient in one of CBCT and CT respectively. **c** shows the DVH of the (**a**) (*solid line*) and (**b**) (*dash line*). **d** displays the dose difference between the (**a**) and (**b**)

**Fig. 4:**
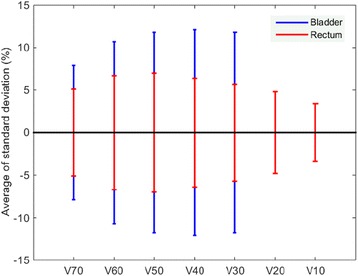
The average of standard deviation of the V_70Gy_, V_60Gy_, V_50Gy_, V_40Gy_ and V_30Gy_ of the bladders and the V_70Gy_, V_60Gy_, V_50Gy_, V_40Gy_, V_30Gy_, V_20Gy_ and V_10Gy_ of the rectum for the all 19 patients

Figure 3(a)–(b) represent dose distribution of the same transversal plane of the same patient in one of CBCT and CT, respectively. Figure 3(d) displays the dose difference between Fig. 3(a) and (b). The dose difference area can be seen on Fig. 3(d) because of the air bubbles which appeared on the CBCT images. The major dose differences which occur around the patient surface are mainly caused by position error and deformation that occurred between fractional CBCT and planning CT. Because the volume of the rectum varies less than the bladder, as shown in Fig. 4, the results from the 19 patients demonstrate that the standard deviation of V_70Gy,_ V_60Gy,_ V_50Gy,_ V_40Gy_ and V_30Gy_ of the bladders are bigger than those of the rectum for all of the patients except for two. In six of the 19 patients, mean V_70Gy_ of the CBCTs are >20 %.

### Dose and volume relationship

**Fig. 5:**
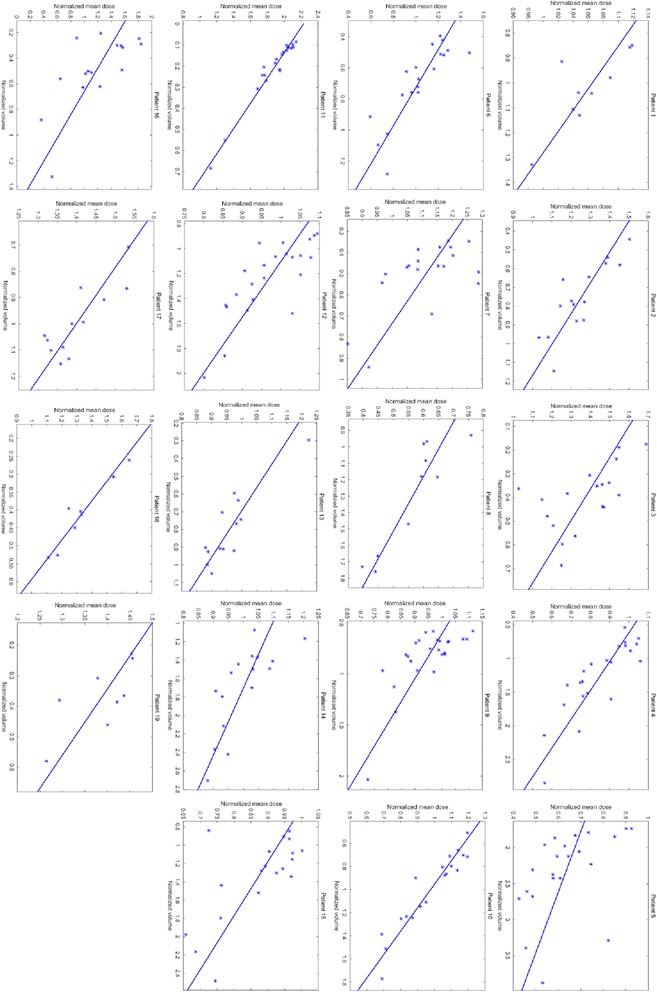
The regression analysis between the normalized mean dose and the normalized volume for each patient’s bladder

Figure 5 displays the regression analysis between the normalized mean dose and the normalized volume for each patient’s bladder. The result of the linear regression analysis shows that the mean dose of the bladder would increase while the volume of the bladder shrinks (all with *p* <0.05), but find no correlation for the rectum (seven patients *p* <0.05, five of them show positive correlation). On average, the slope of the linear relationship between the mean dose and the volume of the bladder is 0.56 ± 0.49, which means if the bladder volume is increased by 10 %, the mean dose will reduce by 5.6 % (±4.9 %).

## Discussion

Previous works showed that the position and shape of the bladder and rectum vary throughout the course of prostate radiotherapy treatment [14–16]. The application of CBCT can improve the accuracy of radiation therapy in target location and dose delivered [17, 18]. In this study, we used CBCT for patient dose recalculation and organs at risk evaluation. Although patient setup error can be corrected through the rigid registration, deformations like the air bubbles in the rectum and the volume variance of the bladder appear to be more important. Fortunately, these deformations only affect the dose distribution slightly in the central area of the patient. The maximum dose difference between initial treatment plan and recalculated dose for the CBCT in the central area is no more than 3.50 Gy, about 5.1–4.2 % of the prescription dose (64.8–79.2 Gy) to the PTV. Therefore, the CTV dose coverage would be sufficient if the CTV does not move out of the PTV area (projected from the original planning CT). It’s necessary to start to monitor the rectum and bladder filling at the treatment planning CT. If rectum and bladder filling are not sufficient, the treatment delivery should be rejected.

The most significant variation in dose area is caused by the air bubbles, which expands the rectum closer to the target. This is one major reason for variations which appeared in rectum dose-volume value. Moreover, the image quality of CBCT suffers from the air bubbles, which blurs the boundary of the rectum and causes uncertainty both on contouring and dose evaluation. Padhani et al. had subjectively graded 5 levels of rectal air/fecal distension and found the moderate and large grades, similar to the cases with air bubbles in this study, were the most likely to produce rectal movements [19]. He suggested that patients should be advised to empty their rectum prior to radiotherapy to reduce rectal distension. To increase the accuracy of the dose distribution, the patients should empty the rectum before radiotherapy treatments.

Bladder shrinkage and expansion are two key factors for dose variances, but are hard to control, especially in cancer centers without careful bladder filling exam. This study found that most patients’ bladder volume varied during treatment. What’s more, bladder volumes of three patients were all smaller than their original state, while in one patient the opposite case was true. Perhaps some patients may not feel comfortable holding urine and did not realize the importance of drinking water to keep the bladder full, so they did not always drink water. Furthermore, some patients may be too nervous and therefore drank more water, making the bladder full. In our hospital, there are too many patients that need to be treated, so physicians and technicians may not always be able to relax the patient or confirm whether the patient drink water before treatment. Patients could not keep the bladder volume constant during each fraction treatment while the air bubbles only existed in a few CBCTs during the treatment course, which might result in the volume of the bladder vary more than the rectum in this study. An increase of 10 % of bladder volume will cause to a 5.6 % reduction of mean dose. This indicates that some new method with quick bladder volume evaluation will be useful for ensuring the accurate dose delivery and sparing the bladder. As Fig. 3 shows, the shape of the rectum can experience irregular changes because of the air bubbles which can decrease the chances of finding a statistical association between the rectum volume and the mean dose.

There are several studies which have sought to define normal tissue dose constraints based on retrospective analyses of dose-volume and toxicity relationships. Pederson et al. found that the rate of Grade 2+ gastrointestinal (GI) toxicity were correlated to V_70Gy_, V_65Gy_ and V_40Gy_ of the rectum. Freedom from Grade 2+ GI toxicity at 4 years was 100 % for men with rectal V_70Gy_ < 10 %, V_65Gy_ < 20 %, and V_40Gy_ < 40 %; 92 % for men with rectal V_70Gy_ < 20 %, V_65Gy_ < 40 %, and V_40Gy_ < 80 %; and 85 % for men exceeding these criteria [20]. Another study showed that the risk of chronic rectal toxicity Grade >= 2 for rectal with V_70Gy_ < 15 % was 9 %, V_70Gy_ between 25 and 40 % was 18 % and V_70Gy_ >40 % was 25 % [21]. They all pointed out that the higher the rectal volume receiving high doses (>40 Gy), especially 70 Gy, the higher the probability that the patient would get Grade 2+ GI. This suggests that when using CBCT to improve the accuracy of the target location, we should also consider keeping the rectum from the high dose area in order to reduce the risk of Grade 2+ GI. As for the bladder, only a few studies found dose-volume relationships associated with the risk of GU toxicity [22]. Perhaps the changes in bladder filling cause changes in bladder DVH parameters which make the establishment of a relationship between the dose-volume and GU toxicity difficult. But it is still important to reduce the volume of bladder which enters the high dose area when using the CBCT to set the patient’s position.

In this study, we only focused on dose verification of the bladder and the rectum for those treated patients. Our results suggest that the current patient instruction with only drink a glass of water is not sufficient to ensure the bladder filling in a busy center. Pretreatment CBCT imaging or quick bladder volume evaluation tool is required for accurate treatment delivery. Especially for the quick bladder volume evaluation tool without additional imaging dose and patient setup time will be a good option for busy center.

## Conclusion

This study used daily cone-beam CT to evaluate the volume changes of bladder and rectum and evaluate the dosimetric changes of bladder and rectum. Our results demonstrate that the volume change of the bladder is more significant than changes in rectal volume for prostate cancer patients. The volume changes of the rectum are not significant except for the air bubbles in the rectum, but bladder volume variations will cause bladder dose changes proportionately. The mean dose received to the bladder will decrease with enlarging the volume. In addition, the CTV will still be covered by the isodose of 95 % of prescription dose during the treatment course.

## Abbreviations

CBCT, cone-beam CT; CTV, clinical target volume; IMRT, intensity modulated radiotherapy; PTV, planned target volume; ROI, region-of-interest; RT, radiotherapy; VMAT, volumetric modulated arc therapy
